# Effect of janus kinase inhibitors and methotrexate combination on malignancy in patients with rheumatoid arthritis: a systematic review and meta-analysis of randomized controlled trials

**DOI:** 10.1186/s13317-021-00153-5

**Published:** 2021-04-28

**Authors:** Vinod Solipuram, Akhila Mohan, Roshniben Patel, Ruoning Ni

**Affiliations:** Department of Internal Medicine, Ascension Saint Agnes Healthcare, 900S Caton Ave, Baltimore, 21229 USA

**Keywords:** Rheumatoid arthritis, Tofacitinib, Baricitinib, Upadacitinib, Filgotinib, Peficitinib, Decernotinib, Jak inhibitors, Methotrexate

## Abstract

**Background:**

Rheumatoid arthritis (RA) is a systemic autoimmune disease. The combination therapy of methotrexate (MTX) and Janus kinase inhibitor (JAKi) is commonly used. Patients with RA are at increased risk of malignancy, however, it remains unclear whether the combination therapy is associated with a higher risk.

**Objective:**

To assess the malignancy risk among patients with RA receiving combination therapy of JAKi and MTX compared to MTX alone.

**Methods:**

PubMed, Cochrane and Embase were thoroughly searched for randomized controlled trials (RCTs) in patients with RA receiving JAKi and MTX, from inception to July 2020. Primary endpoints were malignancy events, Non melanomatous skin cancer (NMSC) and malignancy excluding NMSC and secondary endpoints were serious adverse events (SAE), deaths. Risk ratio (RR) and 95% CI were calculated using the Mantel–Haenszel random-effect method.

**Results:**

659 publications were screened and 13 RCTs with a total of 6911 patients were included in the analysis. There was no statistically significant difference in malignancy [RR = 1.42; 95% CI (0.59, 3.41)], neither NMSC [RR = 1.44 (0.36, 5.76)] nor malignancies excluding NMSC [RR = 1.12 (0.40, 3.13)]. No statistically significant difference between the two groups for SAE [RR = 1.15 (0.90, 1.47)] and deaths [RR = 1.99 (0.75, 5.27)] was found.

**Conclusion:**

The adjunction of JAKi to MTX is not associated with an increased risk of malignancy when compared to MTX alone. There is no increased risk of SAE and deaths when compared to MTX alone in patients with RA.

**Supplementary Information:**

The online version contains supplementary material available at 10.1186/s13317-021-00153-5.

## Introduction

Rheumatoid arthritis (RA) is a systemic autoimmune disease, estimated to affect approximately 0.5% to 1% of population [1]. Patients with RA are predisposed to an increased risk for malignancy, especially malignant lymphomas [2–7], lung cancers [5, 6] and non-melanoma skin cancer [7]. Higher mortality was associated with the presence of cancer, varied by stage of malignancy [8]. Persistent inflammation triggers the development and progression of cancer [9, 10]. It has been well-established that severity of inflammation in RA is positively correlated with the risk of lymphoma [11]. Taking the inflammation into control may reduce the risk of developing malignancy.

Janus kinase (JAK) can initiate lymphocyte activation, function and proliferation via tyrosine phosphorylation of the receptors and downstream signal transducer and activator of transcription (STAT) signaling [12]. JAK-STAT signaling pathways mediate a double-edged sword effect on both antitumor defense and tumor progression [13]. Inhibiting JAK-STAT pathways raises the concern of losing immune cell function to malignancy, as well as proposes the possibility of suppressing tumor formation.

JAK can be divided into 4 types: JAK1, JAK2, JAK3 and Tyrosine kinase 2 (Tyk2), responsive to myriad cytokines. Various Janus kinase inhibitors (JAKi) are being widely investigated in randomized controlled trials (RCTs) and proved efficacy in patients with RA [14]. JAKi consist of tofacitinib, selectively inhibiting JAK1 and JAK3, baricitinib, blocking JAK1 and JAK2, peficitinib, acting on all types of JAK, decernotinib, highly selective for JAK3, upadacitinib and filgotinib, selectively targeting JAK1. Limited evidence demonstrated no statistical difference in malignancy incidences in patients receiving tofacitinib compared to the general population [15]. Since most patients with RA are treated with combinations of traditional disease-modifying antirheumatic drugs (DMARDs), especially methotrexate, it is important to explore the safety profile of these therapies. The role of different JAKi in the risk of malignancy remains undetermined. Even scarce data exists, to see the malignancy outcomes of JAKi when used in combination with methotrexate.

In consideration of the current unclear malignancy risk of JAKi and MTX combination, we sought to explore a potential association between JAKi and MTX combination and malignancies in patients with RA.

## Methods

### Literature search

A systematic search was performed in PubMed, Embase and Cochrane Library without language limitations from inception to July 30, 2020. Search terms included “rheumatoid arthritis”, “tofacitinib”, “baricitinib”, “upadacitinib”, “filgotinib”, “peficitinib”, “decernotinib”, “jak inhibitors”, “methotrexate”. References of the retrieved articles were searched to identify further relevant studies suitable for this meta-analysis. Search strategy is listed in Table 1.

**Table 1:** Search strategies

PubMed search strategies			
Search number	Query	Filters	Results
1	(Rheumatoid arthritis) OR (rheumatoid arthritis[MeSH Terms])		149,891
2	Janus kinase inhibitors		5361
3	Tofacitinib		1435
4	Baricitinib		362
5	Upadacitinib		115
6	Peficitinib		59
7	Decernotinib		22
8	Filgotinib		101
9	#2 OR #3 OR #4 OR #5 OR #6 OR #7 OR #8		6367
10	#1 AND #9		972
11	#1 AND #9	Randomized controlled trial	101

Embase search strategies		
No.	Query	Results
#1	Rheumatoid arthritis' OR 'rheumatoid arthritis':ti,ab,kw	233,332
#2	Janus kinase inhibitor' OR 'janus kinase inhibitor':ti,ab,kw	3663
#3	Tofacitinib' OR 'tofacitinib':ti,ab,kw	4688
#4	Baricitinib' OR 'baricitinib':ti,ab,kw	1216
#5	Upadacitinib' OR 'upadacitinib':ti,ab,kw	419
#6	Peficitinib' OR 'peficitinib':ti,ab,kw	155
#7	Decernotinib' OR 'decernotinib':ti,ab,kw	122
#8	Filgotinib' OR 'filgotinib':ti,ab,kw	403
#9	#2 OR #3 OR #4 OR #5 OR #6 OR #7 OR #8	8061
#10	#1 AND #9	3049
#11	#10 AND 'randomized controlled trial'/de	400

Cochrane search strategies		
ID	Search hits	Search results
#1	MeSH descriptor: [Arthritis, Rheumatoid] explode all trees	6056
#2	MeSH descriptor: [Janus Kinase Inhibitors] explode all trees	43
#3	(filgotinib): ti,ab,kw (word variations have been searched)	132
#4	(tofacitinib):ti,ab,kw (word variations have been searched)	699
#5	(baricitinib):ti,ab,kw (word variations have been searched)	354
#6	(decernotinib):ti,ab,kw (word variations have been searched)	8
#7	(upadacitinib):ti,ab,kw (word variations have been searched)	196
#8	(peficitinib):ti,ab,kw (Word variations have been searched)	23
#9	#2 OR #3 OR #4 OR #5 OR #6 OR #7 OR #8	1414
#10	#1 AND #9	161

### Eligibility criteria

Two independent authors (VS and AM) screened all titles and abstracts for potential inclusion. Any discrepancy among the selected studies were resolved by a third author (RN). Double-blind RCTs that reported malignancy events in adult patients with RA receiving the combination of methotrexate with any JAKi, with methotrexate in the control arm. Studies that did not report malignancy outcomes were excluded. Exclusion criteria included reviews, editorials, letters, observational studies, non human studies. Long term extension studies with single arms and trials involving other biologic DMARDs were excluded as well.

### Data extraction and quality assessment

Two independent investigators (VS and AM) performed the search and relevant studies were selected based on the inclusion and exclusion criteria. VS and AM extracted the data using a predefined data abstraction form: age, sex, duration of rheumatoid arthritis, mean number of tender and swollen joints, length of follow-up. Patient-years were calculated based on the extracted data. Any discordance between these two authors was resolved by the third author (RN). The Cochrane quality assessment tool for RCTs was used to assess risk of bias [16].

### Outcomes of interest

The primary endpoints of interest were the incidence of malignancy, non melanomatous skin cancers (NMSC) and malignancies excluding NMSC. The secondary endpoints were incidence of serious adverse events (SAE) and deaths.

### Statistical analysis

Extracted data were combined using Review Manager (RevMan) software (Cochrane collaboration) Version 5.4. Risk ratio (RR) of malignancies, NMSC, malignancies excluding NMSC, SAE, death was calculated with 95% confidence intervals (CIs) based on Mantel–Haenszel random-effect method. I^2^ was used to evaluate heterogeneity among the studies (< 25% considered low heterogeneity and > 50% considered significant heterogeneity). Publication bias was assessed via the funnel plot for the primary endpoint. Sensitivity analysis was performed by leave-one-out method.

Results were reported according to the Preferred Reporting Items for Systematic Reviews and Meta-Analyses Protocol (PRISMA-P) 2015 statement [17]. The protocol for this systematic review and meta-analysis was registered with PROSPERO (CRD42020201473).

## Results

**Fig. 1:**
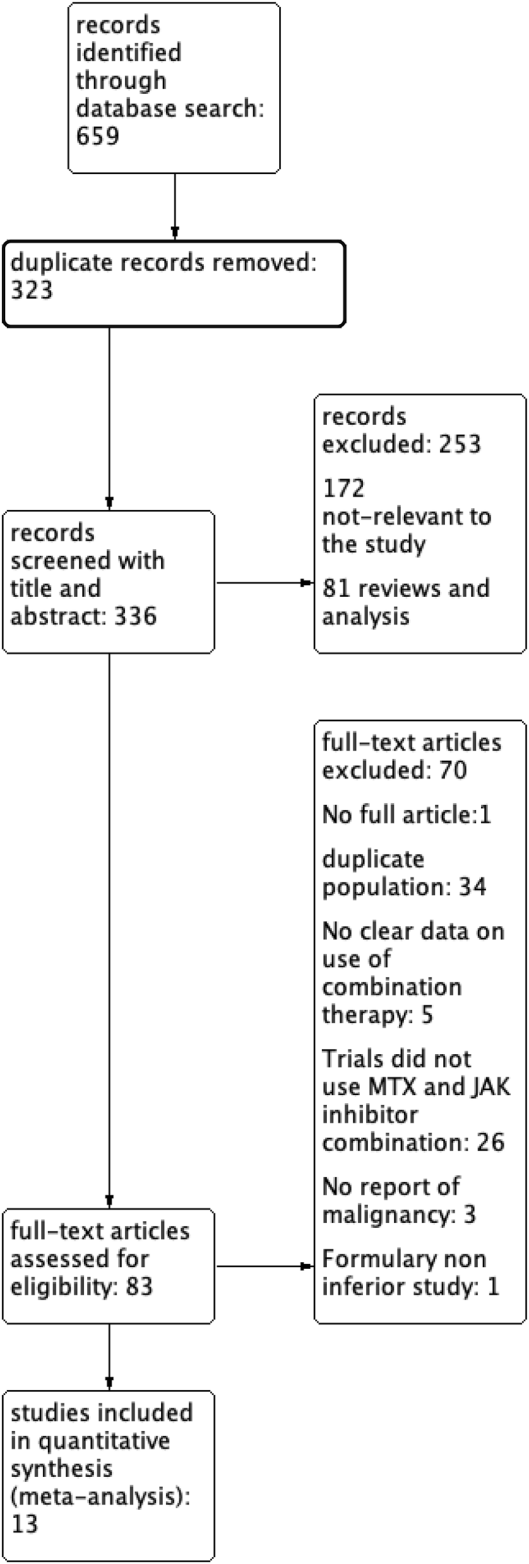
Flow chart of included randomized controlled trials

A total of thirteen RCTs comprising 6911 patients met the inclusion criteria [18–30], as summarised in Fig. 1, with 2377 patients in the MTX group and 4534 patients in the combination of MTX and JAKi group. Total patient-years in the MTX group and JAKi and MTX combination group were 988 and 3684, respectively. Among all the trials included, 3 trials used tofacitinib, 3 trials for baricitinib, 3 trials for upadacitinib, 2 trials for peficitinib and 1 trial for filgotinib in combination with MTX (Additional files 1, 2, 3, 4, 5, and 6).

### Study characteristics

The baseline characteristics of the patients with RA included in the studies were comparable among the two groups, as summarised in Table 2. The length of follow-up of the trials ranged from 12 weeks to 24 months. The mean age for the JAKi and MTX combination group was 53 ± 11.7 years and 53 ± 11.8 years in the MTX alone group. The average duration of RA was 7.6 years in the MTX alone group and 8.2 years in the JAKi and MTX combination group. The crude incidence rates of malignancies (NMSC/non NMSC) in JAKi and MTX combination group and MTX group were, respectively, 1.086 (0.497/0.651) and 0.709 (0.202/0.409) per 100 patient years.

**Table 2:** Characteristics of included trials

Name of study	Type of study	Study phase	Population	Duration of enrollment	Intervention	Number of patients	Follow up duration	Countries	Number of centers	Treatment arms	Primary outcome	Secondary outcomes
Burmester [18]	Double-blind	Phase 3	RA with IR to TNF inhibitors	October, 2009 to March 2011	Tofacitinib and placebo	399	6 months	13 countries including North America, Latin America and Europe	82	Tofacitinib 5 mg, 10 mg BID vs. placebo along with MTX	ACR 20, HAQ-DI	DAS28-ESR
Fleischman [20]	Double-blind	Phase 3/long term	RA with IR to MTX	NA	Upadactinib, placebo, adalimumab	1629	48 weeks	41	286	Upadactinib 15 mg, adalimumab 40 mg, placebo	DAS28-CRP, ACR20, inhibition of radiographic progression	DAS28-CRP mean change of DAS28-CRP, HAQ DI, SF-36, PCS, FACIT-F, CDAI < 10
Fleischman [19], RA-BEGIN	Double-blind	Phase 3	RA with no or minimal csDMARDs and naive to bDMARDs	01/13–08/14	Barictinib, MTX or combination of Barictinib and MTX	588	52 weeks	18 counries	198	Baricitnib 4 mg, Baricitinib 4 mg + MTX	Noninferioritiy of baricitnib monotherapy to MTX monotherapy by ACR20 at 24 weeks	Superiority comparision by ACR 20, HAQ-DI, SDAI, DAS28-CRP, vdH-mTSS
Genovese [21]	Double-blind	Phase 2b	RA with IR to MTX	03/14–07/15	Upadacitinib, placebo	300	12 weeks	16	63	Upadactinib 3 mg bid, 6 mg bid, 12 mg bid, 18 mg bid, 24 mg q day, placebo bid	ACR20	ACR50, ACR70, DAS28-CRP, CDAI
Van der Hejide [25] ORAL SCAN	Double-blind	Phase 3	Active RA with IR to MTX	NA	Tofacitinib vs. placebo	797	24 months	NA	NA	Tofacitinib 5 mg, 10 mg BID vs. placebo	ACR20, ACR50, ACR70, mean changes in DAS28-ESR, CDAI, SDAI, HAQ-DI	NA
Kivitz [22]	Double blind	Phase 2b	Modearte to severe RA with IR to MTX	NA	Peficitinib	378	12 weeks	(8) Usa, poland, hungary, Czech republic, Mexico, Bulgaria, Belgium, Colombia	43	Peficitinib 25, 50, 100, 150 mg	ACR20 using CRP at 12 weeks	ACR50, ACR70, DAS28-CRP, CDAI
Kremer [23]	Double blind	Phase 2b	RA with IR to MTX	10/13–07/15	Upadactinib, placebo, adalimumab	276	12 weeks	10 (North america, Europe, Australia)	123	Upadacitnib 3 mg, 6 mg, 12 mg, 18 mg	ACR20	ACR50, ACR70, Low disease points/remission by DAS28-CRP, CDAI, Change in DAS28-CRP, ACR core set changes, MCID of HAQ DI
Li [24]	Double blind	Phase 3	RA with IR to MTX	NA	Baricitinib, placebo	290	52 weeks	3 (China, Brazil, Argentina)	30	Baricitinib 4 mg	ACR 20 at 12 weeks	HAQ-DI, DAS28-CRP, remission and LDA, SDAI, CDAI, ACR50, ACR70
Takeuchi [26] RAJ4	Double blind	Phase 3	RA pt with IR to MTX	July 2014-Nov 2017	Peficitinib	519	52 weeks	Japan	161	Peficitinib 100 mg, 150 mg	ACR20 at 12 weeks/ET, baseline change in mTSS at 28 weeks/ET	ACR20/50/70 response, DAS28-CRP, DAS28-ESR, CRP, ESR, PGA, TJC68, SJC66, CDAI, SDAI
Tanaka [27]	Double-blind	Phase 2b	pt with moderate to severe RA on MTX	11/11–12/13	Baricitinib, placebo	145	12 weeks	Japan	24	Baricitinib 1 mg, 2 mg, 4 mg, 8 mg	ACR20 at 12 weeks/ET, baseline change in mTSS at 28 weeks/ET	ACR50, ACR70, ACR core components, DAS28-ESR, DAS28-CRP, SDAI, EULAR28
Taylor [28]	Double-blind	Phase 3	pt with RA on MTX	11/12–09/14	Baricitinib 4 mg, adalimumab 40q2wk	1307	52 weeks	26	281	Baricitinib 4 mg, adalimumab 40q2wk	ACR20 at 12 weeks,	mTSS score at 24 weeks, HAQDI, DAS28-CRP, SDAI, PRO at week 12
Vollenhoven [29] ORAL standard study	Double blind	Phase 3	RA pt with IR to MTX	01/09–02/11	Tofacitinib, adalimumab, placebo	717	12 months	Worldwide	115	Tofacitinib 5 mg, 10 mg twice daily, 40 mg of adalimumab q2wks, placeebo	ACR20 reduction in tender and swollen joints at 6 months, 3/5ACR components, HAQ-DI at 3 months, DAS28-ESR	ACR20, ACR50, ACR70 with respect to tender and swollen joints and HAQ-DI
Westhovens [30] DARWIN 1	Double blind	Phase 2b	Active RA with insufficient response to MTX	July 2013 to May 2015	Filgotinib vs. placebo in combination with MTX	594	24 weeks	21 (North and South America, Europe, Asia, Australia)	106	Filgotinib 50 mg, 100 mg, 200 mg once or twice daily vs. placebo	ACR20 at 12 weeks	ACR20, ACR50, ACR70, ACR-N, DAS28-CRP, LDA/remission, EULAR response, EULAR remission, CADI, SDAI

*ACR* American college of rheumatology,* CRP* C-reactive protein,* CDAI* clinical disease activity index,* DAS28* disease activity score 28,* ESR* erythrocyte sedimentation rate,* EULAR* European League Against Rheumatism,* FACIT-F* functional assessment of chronic illness therapy fatigue scale,* HAQ-DI* health assessment questionnaire disability index,* IR* inadequate response,* LDA* low disease activity,* MCID* minimum clinically important difference,* MTX* methotrexate,* NA* not available,* PCS* physical component score,* PGA* physician’s global assessment of disease activity, *PRO* patient reported outcomes,* RA* rheumatoid Arthritis,* SF-36* short-form 36,* SDAI* simplified disease activity index,* SJC* swollen joint count,* TNF-alpha* tumor necrosis factor-alpha,* TJC* total joint count,* vdH-mTSS* van der Heijde-modified total sharp score

### Primary outcomes

**Fig. 2:**
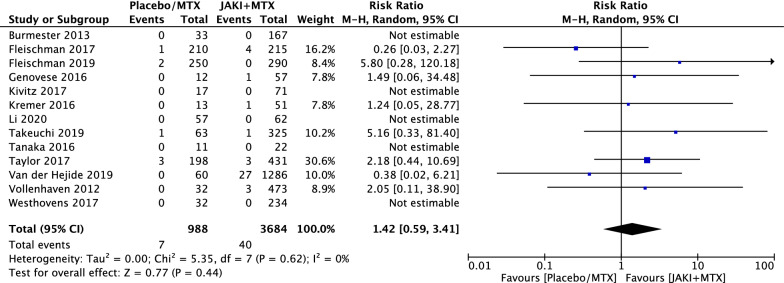
Relative risks (RRs) of all malignancies in patients with rheumatoid arthritis (RA) treated with Janus kinase inhibitors (JAKi) and methotrexate (MTX) compared with MTX alone in randomised controlled trials (RCTs) using the Mantel–Haenszel (M–H) random-effect method

**Fig. 3:**
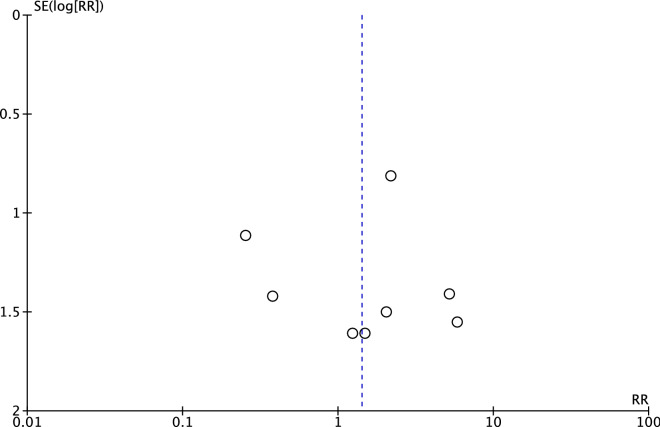
Funnel plots for the meta-analysis of occurrence of all malignancies among JAKi and MTX versus MTX

A total of forty patients suffering from malignancies were reported in the JAKi and MTX group among 3684 patient-years and seven malignancy events in the MTX group among 988 patient-years. There was no statistically significant difference in malignancy events (RR = 1.42; 95% CI 0.59 to 3.41, p = 0.44) between the combination group and the control group, seen in Fig. 2. We found a relatively low level of heterogeneity across all included RCTs (χ^2^ = 5.35, df = 7, p = 0.62, I^2^ = 0%). For the Mantel–Haenszel random methods, funnel plot showed no evidence of publication bias in all comparisons in Fig. 3.

**Fig. 4:**
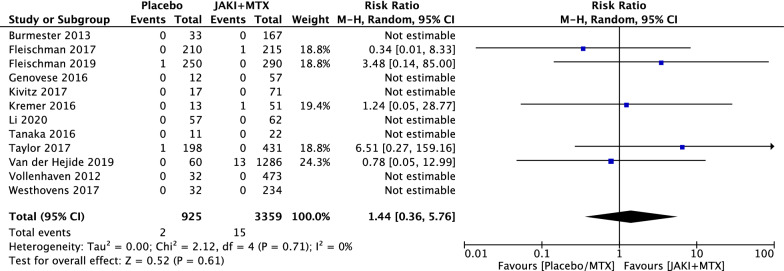
RRs of non melanomatous skin cancer (NMSC) in patients with RA treated with JAKi and MTX compared to MTX alone in RCTs using the M–H random-effect method

**Fig. 5:**
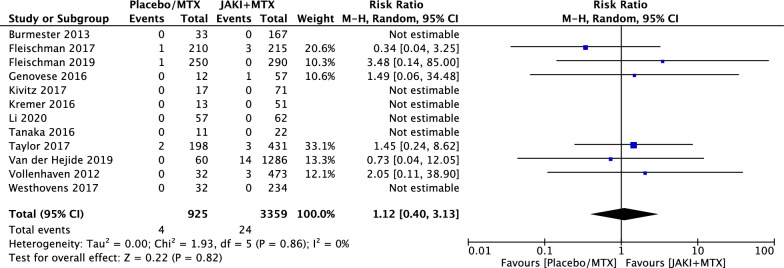
RRs of malignancies excluding NMSC in patients with RA treated with JAKi and MTX compared to MTX alone in RCTs using the M–H random-effect method

Considered separately, statistical differences remained undetectable for non melanomatous skin cancers (NMSC) (RR = 1.44; 95% CI 0.36 to 5.76, p = 0.61) (Fig. 4) and malignancies excluding NMSC (RR = 1.12; 95% CI 0.40 to 3.13, p = 0.82) (Fig. 5). Among 40 malignancy events in the JAKi and MTX combination group, 15 (37.5%) were NMSC and 24 (60%) were malignancies excluding NMSC. Among 7 malignancy events reported in MTX along group, 2 (28.5%) were NMSC and 4 (57%) were malignancies excluding NMSC. One malignancy in each group was not reported in detail. Among solid tumors for the MTX and JAKi combination group, the most common types were cervical cancer in 6 patients (25%), lung cancer in 5 patients (21%), breast cancer in 4 patients (17%) and ovarian cancer in 1 patient. Two cases of non-Hodgkin’s lymphoma and two cases of melanoma were reported.

### Secondary outcomes

**Fig. 6:**
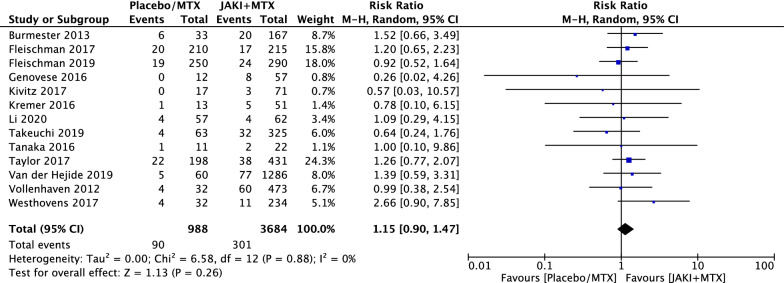
RRs of safety adverse events (SAE) in patients with RA treated with JAKi and MTX compared to MTX alone in RCTs using the M–H random-effect method

**Fig. 7:**
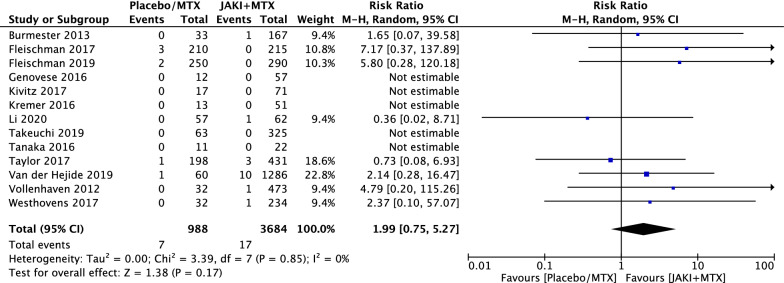
RRs of deaths in patients with RA treated with JAKi and MTX compared to MTX alone in RCTs using the M–H random-effect method

Although more serious adverse events and deaths were reported in the combination therapy group, there was no statistically significant difference between the two groups with RR = 1.15 (95% CI 0.90 to 1.47, p = 0.26) in SAE (Fig. 6) and RR = 1.99 (95% CI 0.75 to 5.27, p = 0.17) in deaths (Fig. 7).

### Sensitivity analysis

The sensitivity analysis was performed for malignancy outcomes with leave one out method and did not show evidence of bias, as summarised in Table 3.

**Table 3.** Sensitivity analysis by leave-one-out method

Study excluded	Malignancy	NMSC	Malignancy excluding NMSC	SAE	Death
Burmester [18]	1.42 (0.59, 3.41)	1.44 (0.36, 5.76)	1.12 (0.40, 3.13)	1.12 (0.87, 1.45)	2.03 (0.73, 5.64)
Fleischman [19]	1.97 (0.75, 5.16)	2.01 (0.43, 9.35)	1.53 (0.48, 4.83)	1.14 (0.87, 1.49)	1.70 (0.61, 4.77)
Fleischman [20]	1.24 (0.50, 3.12)	1.18 (0.25, 5.47)	0.99 (0.33, 2.91)	1.21 (0.92, 1.59)	1.76 (0.63, 4.92)
Genovese [21]	1.41 (0.56, 3.53)	1.44 (0.36, 5.76)	1.09 (0.37, 3.21)	1.16 (0.91, 1.49)	1.99 (0.75, 5.27)
Van der Heijde [25]	1.64 (0.65, 4.14)	1.75 (0.36, 8.61)	1.20 (0.40, 3.61)	1.13 (0.88, 1.46)	1.95 (0.64, 5.89)
Kivitz [22]	1.42 (0.59, 3.41)	1.44 (0.36, 5.76)	1.12 (0.40, 3.13)	1.16 (0.90, 1.48)	1.99 (0.75, 5.27)
Kremer [23]	1.43 (0.57, 3.58)	1.49 (0.32, 6.99)	1.12 (0.40, 3.13)	1.16 (0.90, 1.48)	1.99 (0.75, 5.27)
Li [24]	1.42 (0.59, 3.41)	1.44 (0.36, 5.76)	1.12 (0.40, 3.13)	1.15 (0.90, 1.48)	2.37 (0.85, 6.59)
Takeuchi [26]	1.22 (0.48, 3.09)	Not estimated	Not estimated	1.19 (0.93, 1.54)	1.99 (0.75, 5.27)
Tanaka [27]	1.42 (0.59, 3.41)	1.44 (0.36, 5.76)	1.12 (0.40, 3.13)	1.15 (0.90, 1.48)	1.99 (0.75, 5.27)
Taylor [28]	1.17 (0.41, 3.37)	1.02 (0.22, 4.73)	0.99 (0.28, 3.46)	1.12 (0.84, 1.48)	2.51 (0.85, 7.37)
Vollenhoven [29]	1.37 (0.54, 3.43)	1.44 (0.36, 5.76)	1.03 (0.35, 3.08)	1.16 (0.90, 1.50)	1.82 (0.65, 5.05)
Westhovens [30]	1.42 (0.59, 3.41)	1.44 (0.36, 5.76)	1.12 (0.40, 3.13)	1.10 (0.86, 1.42)	1.95 (0.70, 5.43)

*NMSC* non melanomatous skin cancer, *SAE* serious adverse events

### Risk of bias assessment

**Fig. 8:**
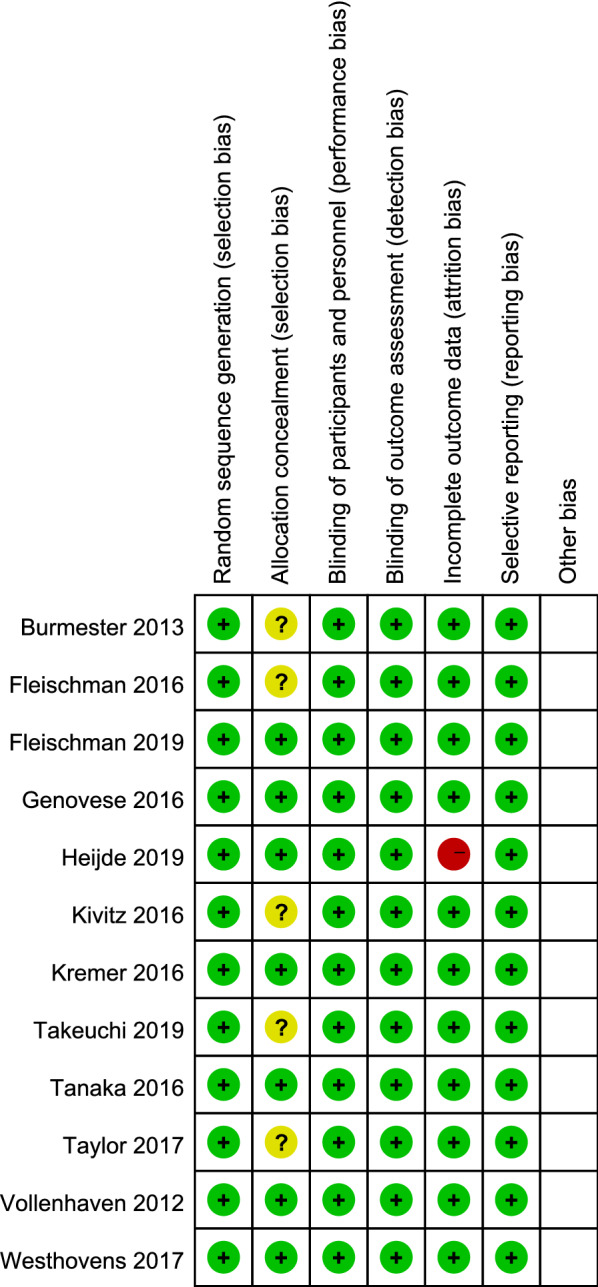
Risk of bias summary

All RCTs (100%) adequately reported the generation of random sequence and 7 RCTs (53.8%) had adequate descriptions of concealed allocation. Blinding of participants, personnel, and outcome assessor was performed in all RCTs. Only 1 RCTs was unable to report the complete outcome. No selective reporting was discovered, seen in Fig. 8.

## Discussion

Patients with RA are predisposed to a higher risk for malignancy [2–7]. It remains not entirely clear if the increased risk of malignancy is primarily related to the pathogenesis of the disease or due to the immunosuppressive therapy. Currently, the combination of JAKi and MTX is widely used in patients with RA. However, whether biologic therapy increases the risk of malignancy has not been well addressed. According to our results, combination therapy of JAKi and MTX did not show any statistically significant increase in the number of malignancies as compared to MTX alone, neither in NMSC nor in malignancies excluding NMSC. Moreover, there are no statistical significant differences that were discovered in the incidence of SAE and mortality among these two groups. The majority of SAE are associated with infections including herpes zoster virus (HZV), urinary tract infection (UTI) and upper respiratory tract infections (URTI). To be noticed, the exposure time of patients with RA to JAKi and MTX is positively correlated to SAE as evidenced by the trials with longer follow up [19, 25, 26]. Among all the trials included, the mortality incidences were similar between patients under JAKi and MTX combination therapy and MTX monotherapy except ORAL SCAN trial [25], in which the combination group had a higher proportion of deaths compared to MTX alone. The deaths were predominantly contributed to multi-organ dysfunction, acute respiratory distress syndrome (ARDS) and major cardiovascular events (MACEs).

To our knowledge, this is the first systematic review exclusively to explore the safety profile, especially from the malignancy perspective, of JAKi and MTX combination therapy.

According to observational studies, patients with RA have an increased risk of malignancy [3, 4, 31]. This is well studied in non-Hodgkin’s lymphoma (NHL), with diffuse large B-cell lymphoma (DLBCL) as the most common type of NHL among patients with RA. These patients are also at increased risk of non-hematologic malignancy such as lungs, kidney, nasopharyngeal carcinoma [32]. The pathophysiology is associated with organ damage from chronic inflammation, genetic mutations, and autoimmune B lymphocyte activation [3, 32]. Viral infection, such as Epstein–Barr virus (EBV) infection may also play a role in the development of B cell lymphoma [33]. It was estimated that patients with RA have approximately 12-fold risk of developing lymphoma and twofold for lung cancer [34]. The risk of developing malignancy is closely related to disease activity [11]. Overtime, the stimulated immune cells from chronic inflammation may undergo malignant transformation, eventually leading to lymphoma. Interestingly, the chronic use of non-steroid anti-inflammatory drugs (NSAIDs) in these patients was associated with a decreased risk of developing colorectal cancer [34].

In terms of treatment, safety profiles, especially the effects on malignancy, should be carefully addressed. Previous studies have shown association between specific therapies and their corresponding risk of malignancy. The role of MTX on the incidence of malignancies remains controversial. Several population based studies did not show any increased risk of malignancies with MTX use compared to baseline risk among patients with RA [35, 36]. MTX may even decrease the risk of lymphoma by suppressing immunologic activation in RA activity but at the same time can increase the risk of other lymphomas [37]. The effects of JAKi on the risk of malignancies still remain unclear. Although genetic variation of JAK-STAT pathway is a known risk factor for malignancy [38], it is unclear whether the use of JAKi would lead to a decreased incidence of malignancies. US Corrona RA registry, a 5-year prospective observational study, did not detect any statistically significant difference in developing malignancy between tofacitinib and other biologic DMARDs [39]. There was no increased risk of malignancy for tofacitinib in patients with RA, when compared to conventional synthetic DMARDs or tumor necrosis factor-α inhibitors per a meta-analysis of observational studies [40]. With the widespread use of JAKi, not only tofacitinib, and MTX among patients with RA, there is accumulating data from multiple RCTs and a definite necessity to explore the safety profile of the combination therapy.

It is important that the effect of dose and exposure time of JAKi and MTX combination on malignancy should be interpreted with caution. Among all the trials included in our study, ORAL SCAN [25] study using tofacitinib, reported the largest number of malignancies. This trial is also distinguished for the longest follow up period of 24 months. However, a definitive conclusion should not be drawn from this single study and more data from long term extension studies is warranted to establish the association between exposure length and incidence of malignancy. Moreover, there is no significant difference in risk of malignancy between high dose and low dose JAKi and MTX.

The results of our secondary analysis are consistent with previous studies, while still with few differences [41]. A previously published meta-analysis also showed no significant increase in the number of malignancies with JAKi treatment but it did not specifically account for the effect of MTX [41]. Safety profile of JAKi and MTX combination is comparable to MTX alone as reported in previous studies. Incidence of SAE and deaths in our analysis were similar to prior published studies. It was reported that higher doses of JAKi were associated with increased SAE, but studies included in our analysis had comparable SAE across different dose ranges of JAKi without large differences [41]. Although not included in our analysis, cardiovascular mortality was also not significantly high in patients receiving JAKi when compared to placebo [42]. Overall JAKi has an acceptable safety profile and combination with MTX did not change it.

There are several limitations in our study and the results should be carefully interpreted. First, the patient-year exposure in the control group was much less compared to the combination group. The increased number of malignancies in the combination group, may be partly related to longer inflammation exposure in the setting of RA. Secondly, long term extension studies were excluded as they were mostly single arm studies without a control group, which could result in potential bias. Last, the majority of malignancies have a latency period and develop over the course of months to years and some trials included in our analysis had relatively limited follow up duration which may underestimate the actual malignancy rate. The strengths of our study is that it included large patient-years to detect any differences among the groups. There was no heterogeneity among the studies included in our analysis and the results of sensitivity analyses are consistent with overall results, proving the robustness of the study.

## Conclusion

Our meta-analysis showed combination therapy of JAKi and MTX did not increase the malignancies in rheumatoid arthritis patients when compared to MTX alone. SAE and deaths are also not significantly different among the two groups. These results have been consistent among all the studies included in the analysis suggesting overall acceptable safety profile of JAKi and MTX combination.

## Supplementary Information

**Additional file 1: Figure S1.** RRs of all malignancy in patients with RA treated with JAKi and MTX compared to MTX alone in RCTs using the M–H random-effect method, subgroup by JAKi and dose.

**Additional file 2: Figure S2.** RRs of all malignancy in patients with RA treated with Tofacitinib and MTX compared to MTX alone in RCTs using the M–H random-effect method.

**Additional file 3: Figure S3.** RRs of all malignancy in patients with RA treated with Baricitinib and MTX compared to MTX alone in RCTs using the M–H random-effect method.

**Additional file 4: Figure S4.** RRs of all malignancy in patients with RA treated with Updacitinib and MTX compared to MTX alone in RCTs using the M–H random-effect method.

**Additional file 5: Figure S5.** RRs of all malignancy in patients with RA treated with Filgotinib and MTX compared to MTX alone in RCTs using the M–H random-effect method.

**Additional file 6: Figure S6.** RRs of all malignancy in patients with RA treated with Peficitinib and MTX compared to MTX alone in RCTs using the M–H random-effect method.

## Data Availability

All relevant data are within the paper.

## Abbreviations

ARDS: Acute respiratory distress syndrome
CI: Confidence interval
DLBCL: Diffuse large B-cell lymphoma
DMARDs: Disease-modifying antirheumatic drugs
EBV: Epstein–Barr virus
HZV: Herpes zoster virus
JAK: Janus kinase
JAKi: Janus kinase inhibitors
MACEs: Major cardiovascular events
MTX: Methotrexate
NHL: Non-Hodgkin’s lymphoma
NMSC: Non melanomatous skin cancer
NSAIDs: Non-steroid anti-inflammatory drugs
PRISMA-P: Preferred Reporting Items for Systematic Reviews and Meta-Analyses Protocol
RA: Rheumatoid arthritis
RCTs: Randomized controlled trials
RR: Risk ratio
SAE: Serious adverse events
STAT: Signal transducer and activator of transcription
Tyk2: Tyrosine kinase 2
URTI: Upper respiratory tract infection
UTI: Urinary tract infection
