# Supplementary figures and images for: Effect of janus kinase inhibitors and methotrexate combination on malignancy in patients with rheumatoid arthritis: a systematic review and meta-analysis of randomized controlled trials
Source: Auto Immun Highlights. 2021 Apr 28;12(1):8. doi: 10.1186/s13317-021-00153-5 (PMC8080865; doi:10.1186/s13317-021-00153-5)

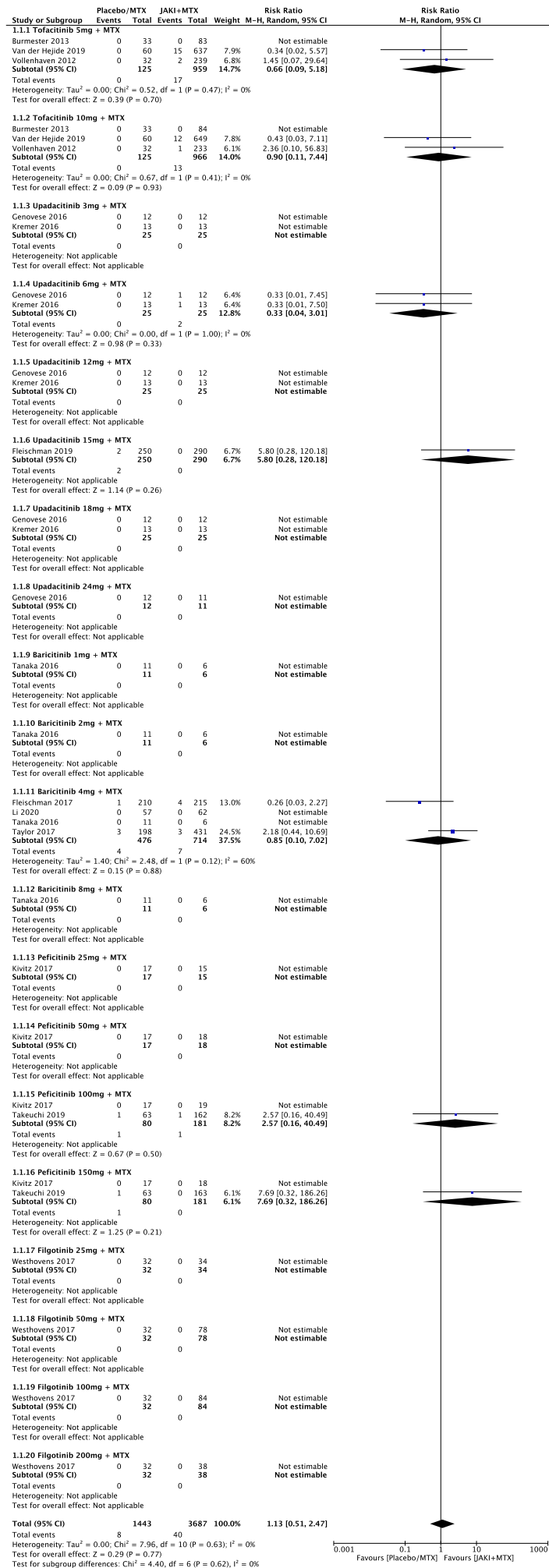

Supplement: Supplementary file 1 — Additional file 1: Figure S1. RRs of all malignancy in patients with RA treated with JAKi and MTX compared to MTX alone in RCTs using the M–H random-effect method, subgroup by JAKi and dose. [file 13317_2021_153_MOESM1_ESM.pdf]

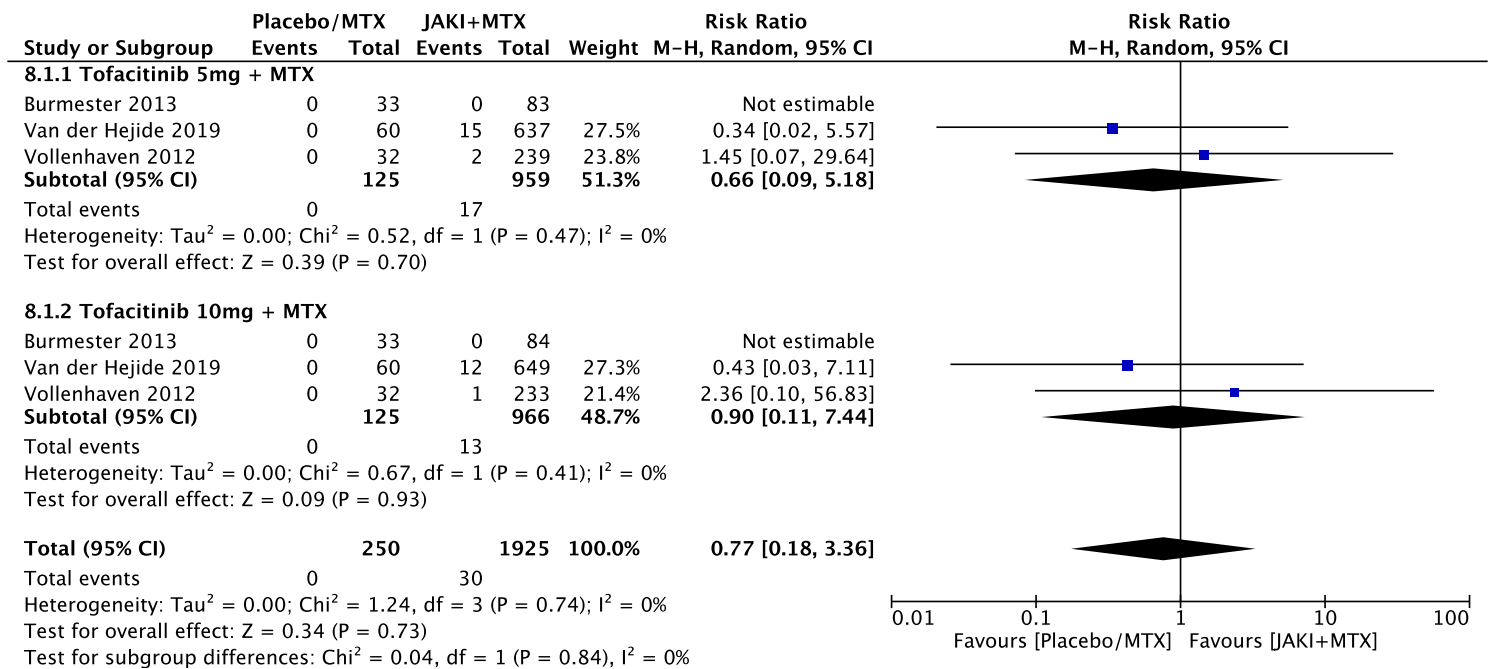

Supplement: Supplementary file 2 — Additional file 2: Figure S2. RRs of all malignancy in patients with RA treated with Tofacitinib and MTX compared to MTX alone in RCTs using the M–H random-effect method. [file 13317_2021_153_MOESM2_ESM.pdf]

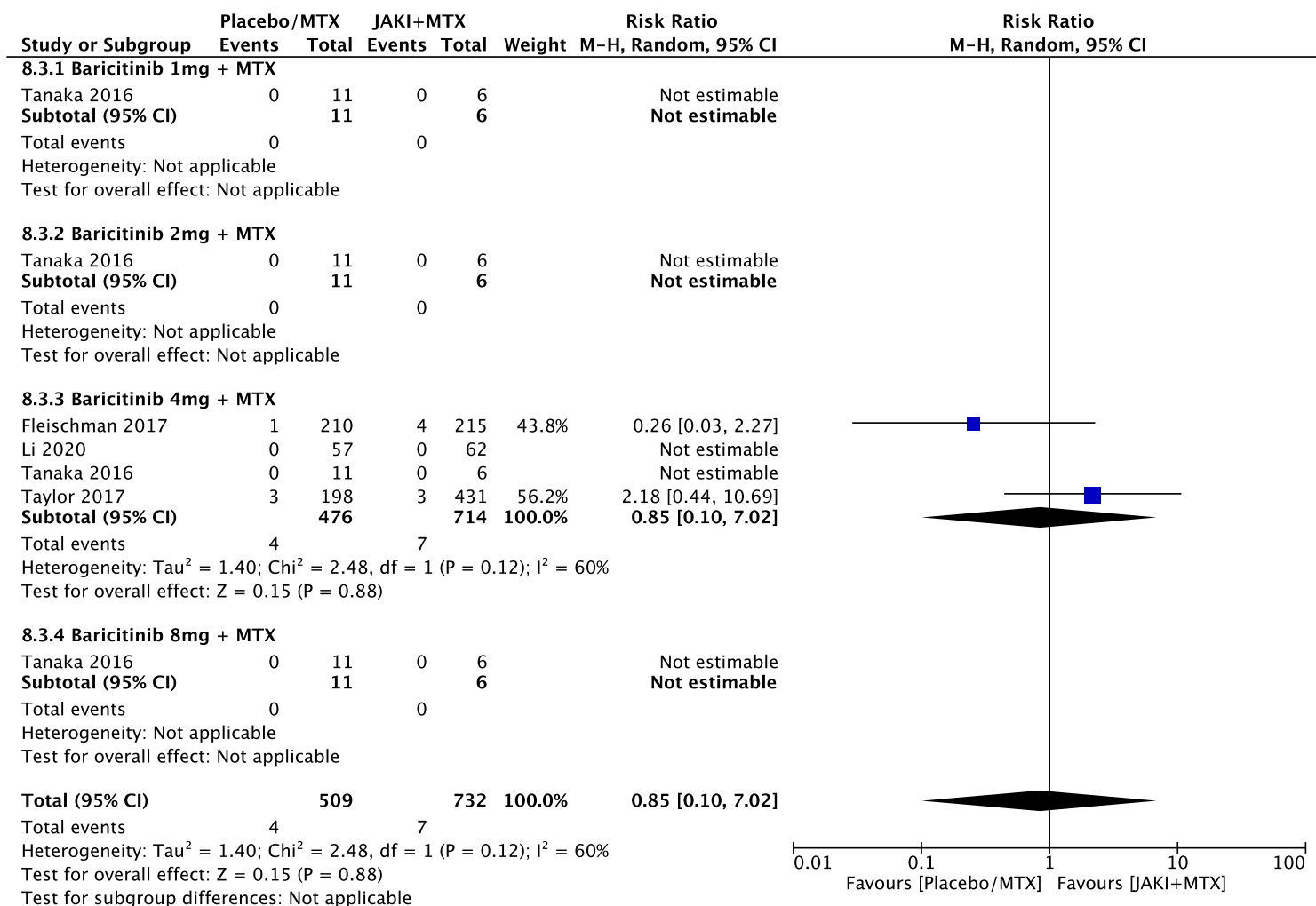

Supplement: Supplementary file 3 — Additional file 3: Figure S3. RRs of all malignancy in patients with RA treated with Baricitinib and MTX compared to MTX alone in RCTs using the M–H random-effect method. [file 13317_2021_153_MOESM3_ESM.pdf]

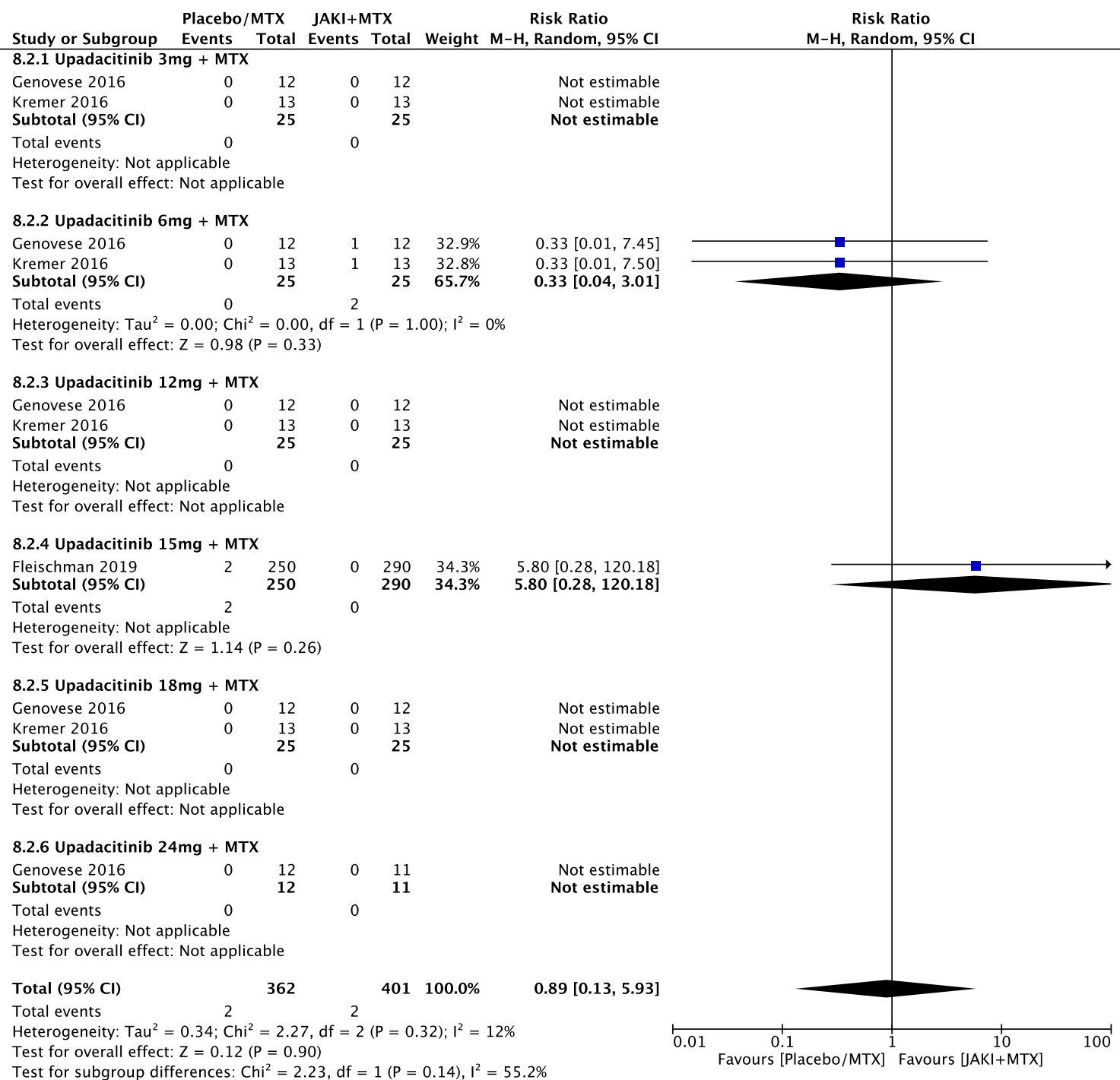

Supplement: Supplementary file 4 — Additional file 4: Figure S4. RRs of all malignancy in patients with RA treated with Updacitinib and MTX compared to MTX alone in RCTs using the M–H random-effect method. [file 13317_2021_153_MOESM4_ESM.pdf]

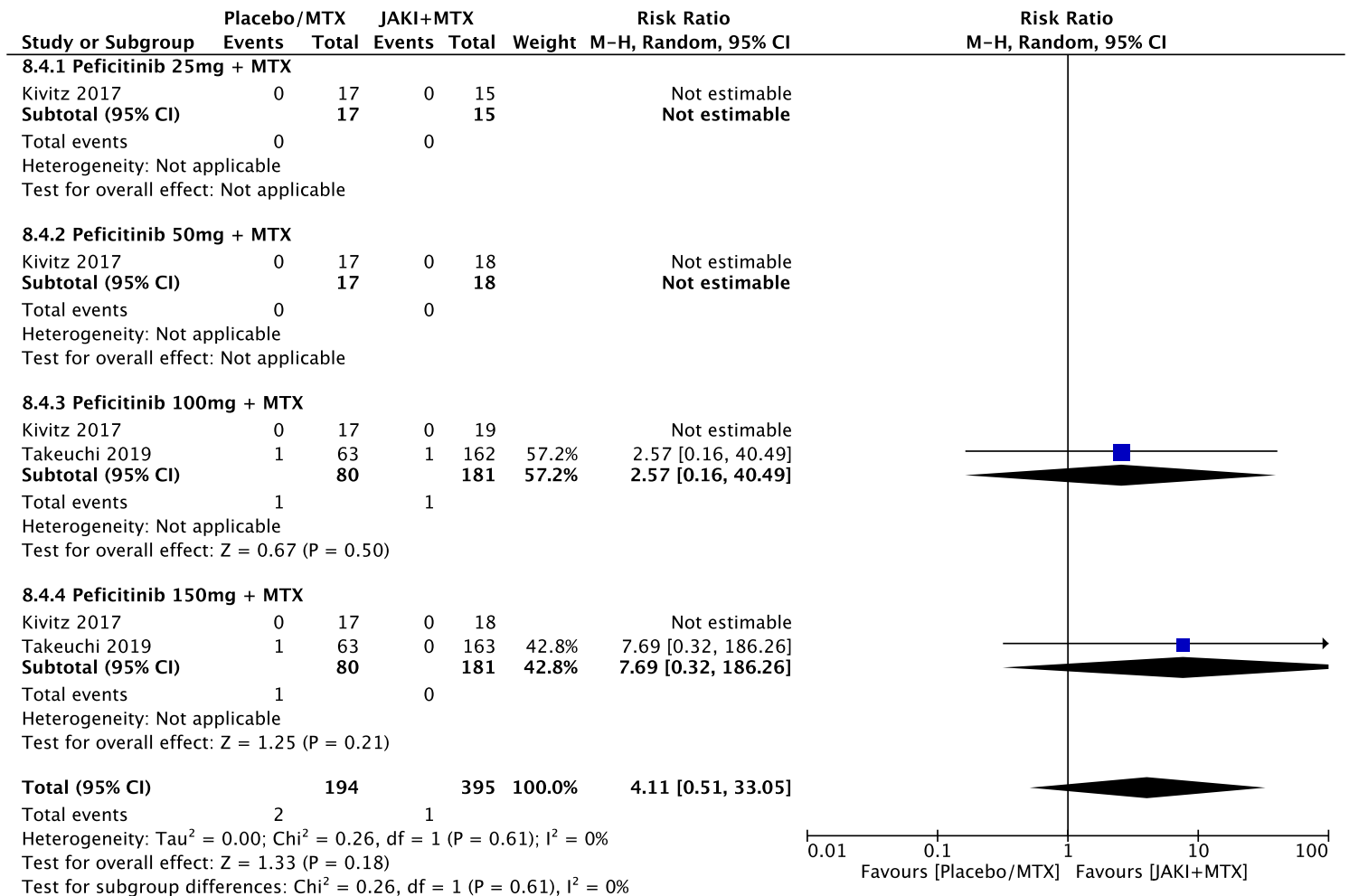

Supplement: Supplementary file 6 — Additional file 6: Figure S6. RRs of all malignancy in patients with RA treated with Peficitinib and MTX compared to MTX alone in RCTs using the M–H random-effect method. [file 13317_2021_153_MOESM6_ESM.pdf]
