# Supplementary material for: Effect of janus kinase inhibitors and methotrexate combination on malignancy in patients with rheumatoid arthritis: a systematic review and meta-analysis of randomized controlled trials
Source: Auto Immun Highlights. 2021 Apr 28;12(1):8. doi: 10.1186/s13317-021-00153-5 (PMC8080865; doi:10.1186/s13317-021-00153-5)

Study or Subgroup	Placebo/MTX Events	Placebo/MTX Total	JAKI+MTX Events	JAKI+MTX Total	Weight	Risk Ratio M-H, Random, 95% CI
**8.5.1 Filgotinib 25mg + MTX**						
Westhovens 2017	0	32	0	34		Not estimable
**Subtotal (95% CI)**		**32**		**34**		**Not estimable**
Total events	0		0			
Heterogeneity: Not applicable						
Test for overall effect: Not applicable						
**8.5.2 Filgotinib 50mg + MTX**						
Westhovens 2017	0	32	0	78		Not estimable
**Subtotal (95% CI)**		**32**		**78**		**Not estimable**
Total events	0		0			
Heterogeneity: Not applicable						
Test for overall effect: Not applicable						
**8.5.3 Filgotinib 100mg + MTX**						
Westhovens 2017	0	32	0	84		Not estimable
**Subtotal (95% CI)**		**32**		**84**		**Not estimable**
Total events	0		0			
Heterogeneity: Not applicable						
Test for overall effect: Not applicable						
**8.5.4 Filgotinib 200mg + MTX**						
Westhovens 2017	0	32	0	38		Not estimable
**Subtotal (95% CI)**		**32**		**38**		**Not estimable**
Total events	0		0			
Heterogeneity: Not applicable						
Test for overall effect: Not applicable						
**Total (95% CI)**		**128**		**234**		**Not estimable**
Total events	0		0			
Heterogeneity: Not applicable						
Test for overall effect: Not applicable						
Test for subgroup differences: Not applicable						

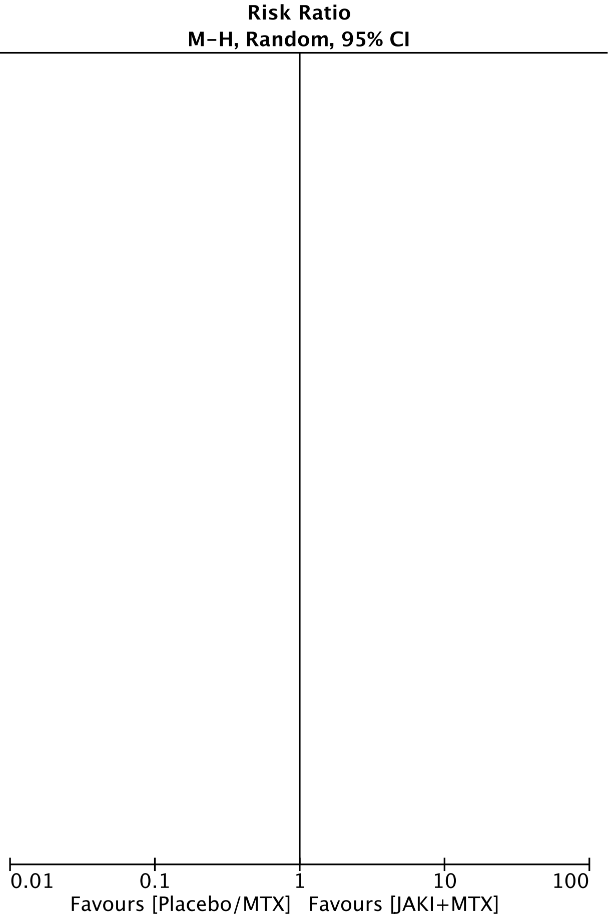

Supplement: Supplementary file 5 — Additional file 5: Figure S5. RRs of all malignancy in patients with RA treated with Filgotinib and MTX compared to MTX alone in RCTs using the M–H random-effect method. [file 13317_2021_153_MOESM5_ESM.pdf]
